# Atrial Fibrillation and Resistant Stroke: Does Left Atrial Appendage Morphology Matter? A Case Report

**DOI:** 10.3389/fneur.2020.592458

**Published:** 2020-11-12

**Authors:** Cristina Sarti, Miroslava Stolcova, Giulia Domna Scrima, Fabio Mori, Ylenia Failli, Donatella Accavone, Silvia Biagini, Costanza Maria Rapillo, Patrizia Nencini, Alessio Mattesini, Carlo Di Mario, Francesco Meucci

**Affiliations:** ^1^NEUROFARBA Department, Neuroscience Section, University of Florence, Florence, Italy; ^2^Stroke Unit, Careggi University Hospital, Florence, Italy; ^3^Structural Interventional Cardiology, Careggi University Hospital, Florence, Italy; ^4^Cardiovascular Diagnostics, Careggi University Hospital, Florence, Italy

**Keywords:** case-report, resistant stroke, left atrial appendage occlusion, atrial fibrillation, anticoagulants, recurrent stroke

## Abstract

**Introduction:** Patients with atrial fibrillation (AF) can experience ischemic stroke despite adequate anticoagulant therapy. The secondary prevention strategy of these so-called “resistant strokes” is empirical. Since about 90% of patients with ischemic stroke due to atrial fibrillation have thrombus in left atrial appendage (LAA) we sought to explore the possibility that resistant stroke could have a LAA morphology resistant to anticoagulants.

**Case Report:** A 77 years old man affected by AF experienced two cardioembolic ischemic stroke while on anticoagulants. The study of LAA showed a windsock-like morphology in the proximal part while distally the LAA presented a cauliflower morphology with a large amount of pectinate muscles and blood stagnation. The precise characteristics of LAA were properly understood integrating images obtained by cardiac CT, transesophageal echocardiography, and selective angiography. A high risky LAA for thrombus formation was diagnosed and its occlusion (LAAO) as an add-on therapy to anticoagulants was proposed and performed. Six month follow-up was uneventfully.

**Conclusion:** The systematic study of LAA in patients with resistant-stroke could help to identify LAA malignant morphology. The efficacy on stroke recurrence of the combined therapy (anticoagulants plus LAAO) is worthy to be tested in randomized trials.

## Introduction

Anticoagulant therapy is the first option for ischemic stroke prevention in patients with atrial fibrillation. However, ~1–2% of patients experience an ischemic stroke while taking anticoagulants (1). An unproperly conducted therapy, such as suboptimal dosage, poor adherence, drugs interactions, or a pathogenesis of stroke not related to atrial fibrillation are possible explanations. If the cardioembolic stroke occurs despite the anticoagulant therapy being well conducted, the event should be classified as a “resistant” stroke, a condition little studied for which the therapeutic approach remains empirical (2).

## Case Description

A 77-year-old man with high blood pressure, diabetes, coronary artery disease, and permanent atrial fibrillation was referred to our Heart-Brain Outpatient Clinic for a second opinion on the best secondary prevention therapy after two previous ischemic strokes.

**Figure 1:**
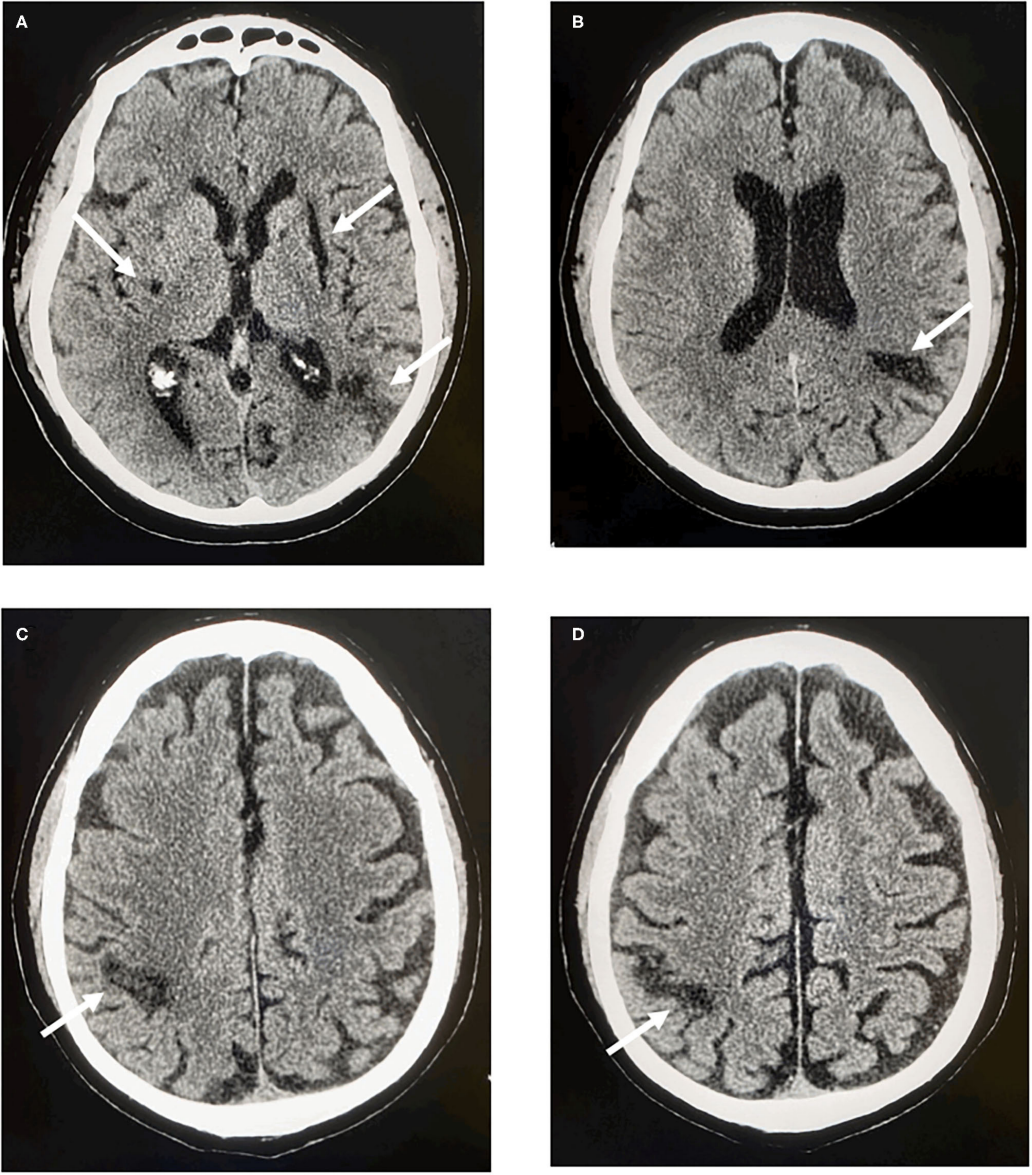
CT scan of the brain. **(A)** Left side: cortico-subcortical temporo-parieto-insular hypodensity. Right side: subcortical lenticular hypodensity. **(B)** Left side: cortico-subcortical parietal hypodensity. **(C,D)** Right side: cortico-subcortical parietal hypodensity.

The first event was characterized by right-sided hemiparesis affecting mainly the arm associated with right hemianopia and hypoesthesia. Neuroimaging revealed a left temporo-parieto-insular cortico-subcortical ischemic lesion (Figures 1A,B).

At the time of the event the patient was taking apixaban 2.5 mg twice daily even though he had normal kidney function and a body weight of 85 kg. Therapy was then changed to dabigatran 150 mg twice daily. Two months later the patient had another ischemic stroke characterized by motor impairment in the left hand associated with a right cortico-subcortical parietal ischemic lesion (Figures 1C,D).

Based on the results of thorough stroke work-up, excluding among others atherosclerotic plaques in cervical and intracranial arteries, aortic-arch atherosclerosis, and occult cancer, atrial fibrillation was considered to be the most likely etiology of patient's recurrent stroke.

Patient reported excellent adherent to anticoagulation therapy. Trough and peak dabigatran plasma levels were within the expected therapeutic range (71 ng/ml, range 61–143; 134 ng/ml, range 117–275, respectively).

The use of concomitant medications that may have altered the dabigatran effect was excluded.

The event was therefore classified as “resistant stroke.”

**Figure 2:**
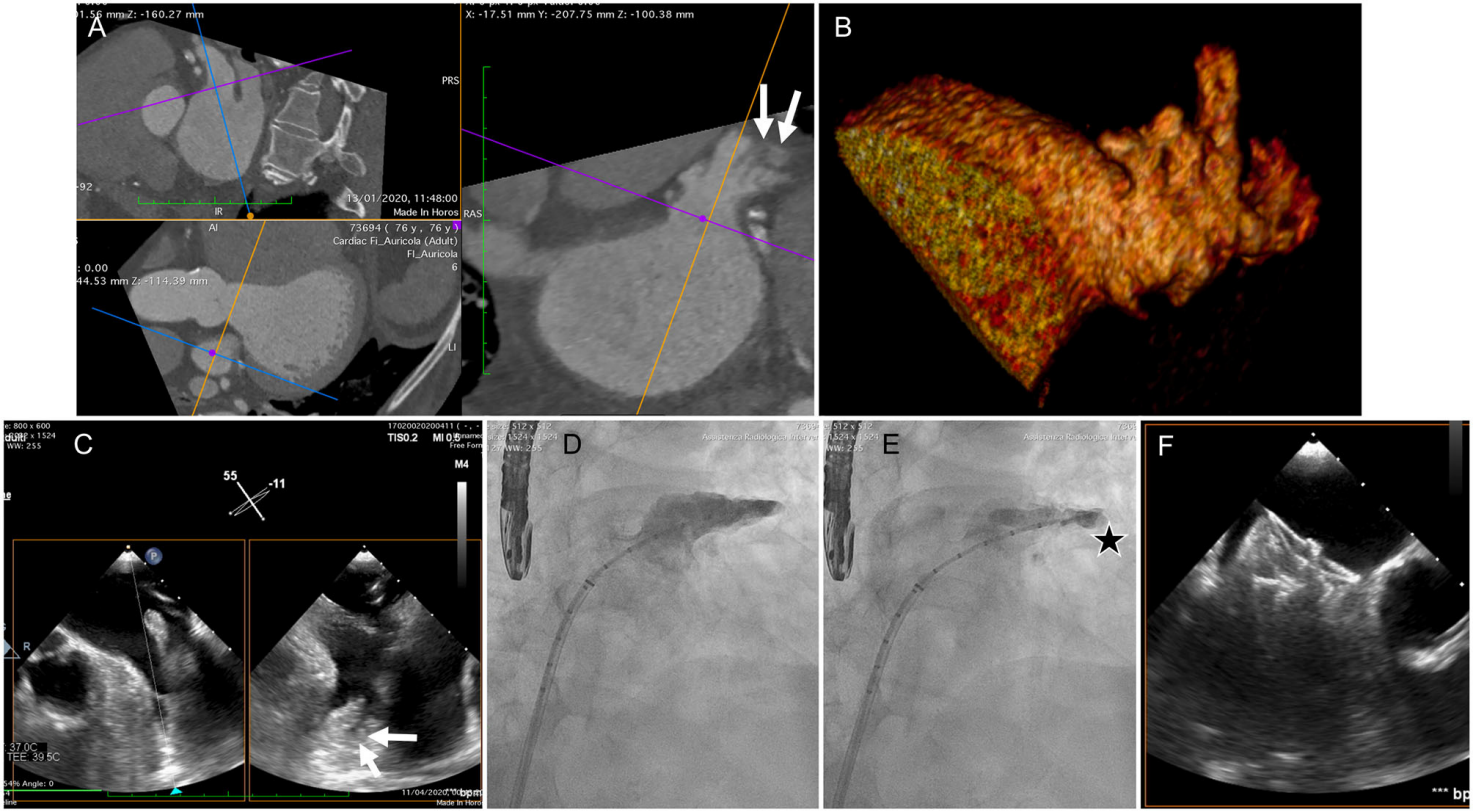
**(A)** CT scan of the LAA. Proximally the LAA presented a single lobe with oval-shaped ostium while distally it divided into multiple lobes, favoring blood stagnation (arrows), configuring a cactus morphology **(B)**. **(C)** Transesophageal echocardiography, performed intraprocedurally revealed a “plus” image in the distal part of the appendage (arrows). **(D)** selective angiography revealed stagnation of dye in the distal lobe, with a slow wash out (**E** – star). **(F)** Final result after successful LAA occlusion with implantation of a 25 mm Amplatzer Amulet device (Abbott, Chicago, IL, USA).

The patient underwent a thorough cardiological study. Cardiac computed tomography (CT) scan was performed to assess the left atrial appendage (LAA). It revealed an oval-shaped LAA with windsock-like morphology in the proximal part (ostium and landing zone) while distally the LAA presented multi-lobated with a large amount of pectinate muscles and a small retro-verse distal lobe with a small amount of blood stagnation (Figure 2A), configuring a cauliflower morphology according to Di Biase criteria (3) (Figure 2B). The mean diameter of the landing zone was 21 mm. The morphology of this LAA was considered to be at high risk for thrombus formation due to its dimensions, distal fragmentation and presence of small lobatures, favoring blood stagnation.

Taking into account the patient's clinical history, the occlusion of LAA as an adjunct therapy to oral anticoagulants was considered and proposed to the patient who, after adequate information, gave his consent.

Transesophageal echocardiography was performed prior to the procedure revealing a “plus” image in the distal part of the appendage suspected of being a clot (Figure 2C). Selective angiography showed stagnation of contrast in the distal lobe, with a slow wash out (Figures 2D,E). The integrated evaluation of the three imaging modalities allowed to attribute the anomalies to a prominent pectinate muscle.

LAA-occlusion was successfully performed with a 25 mm Amplatzer Amulet occluder (Abbott, Chicago, IL, USA) (Figure 2F) and subsequent recovery was uneventful. Patient was discharged with dabigatran 150 mg twice daily and an indication to maintain it lifelong.

The patient was asymptomatic at 6-months follow-up.

## Discussion and Conclusion

About 90% of heart clots in patients with atrial fibrillation develop within the LAA. LAA has a highly variable form and preliminary data suggest an association between LAA morphology and stroke (3, 4). Other LAA features, such as trabeculation, orifice area, LAA flow velocity, seem to identify LAA at high risk of thrombosis (5). One study explored the safety and efficacy of LAAO as secondary prevention in patients with nonvalvular atrial fibrillation who have experienced a stroke/transient ischemic attack despite anticoagulant treatment (6). A recent prospective, observational study on 19 patients explored the efficacy to combine LAAO to anticoagulants (7).

No data are however available on LAA morphology in resistant stroke.

This case highlights that an integrated study with different techniques (CT, echo, angiography) can be of help to identify LAA with high-risk characteristics. The LAA classification in use, limited to the external form, seems too simple and unable to meet this clinical need (3).

In the absence of specific studies focusing on patients with recurrent stroke despite optimal anticoagulation (“resistant” stroke), guidelines are lacking.

Such patients could have a malignant LAA “resistant” to anticoagulants.

A systematic study of LAA morphology may suggest that anticoagulant failure in some of these patients could be caused by an unfavorable form of LAA with high thrombotic risk for which anticoagulants alone may not be sufficient. Consequently, LAA occlusion can be considered not as an alternative but as an adjunct to anticoagulation in selected cases.

The efficacy of this “add-on” combination therapy on stroke recurrence deserves to be tested in randomized trials.

## Data Availability Statement

The raw data supporting the conclusions of this article will be made available by the authors, without undue reservation.

## Ethics Statement

Ethical review and approval was not required for the study on human participants in accordance with the local legislation and institutional requirements. The patients/participants provided their written informed consent to participate in this study. Written informed consent was obtained from the individual(s) for the publication of any potentially identifiable images or data included in this article.

## Author Contributions

CS and FMe: contributed to the clinical part of the study, design and conceptualized study, drafted and revised the manuscript. MS: drafted and revised the manuscript. GDS: contributed to the design of the study and revised the manuscript. FMo: contributed to the clinical part of the study and revised the manuscript. YF, PN, AM, and CDM: revised the manuscript. DA, SB, and CMR: contributed to the clinical part of the study. All authors contributed to the article and approved the submitted version.

## Conflict of Interest

CDM received institutional funding from Edwards, Medtronic, Astra-Zeneca, Daiichi Sankyo, and Shockwave medical. The remaining authors declare that the research was conducted in the absence of any commercial or financial relationships that could be construed as a potential conflict of interest.
